# Evaluation of enhanced recovery after surgery program components implemented in laparoscopic appendectomy: prospective randomized clinical study

**DOI:** 10.1038/s41598-020-67591-5

**Published:** 2020-07-01

**Authors:** Taras Nechay, Alexander Sazhin, Svetlana Titkova, Alexander Tyagunov, Mikhail Anurov, Kirill Melnikov-Makarchuk, Anton Tyagunov

**Affiliations:** Pirogov Russian National Research Medical University, Moscow, Russia

**Keywords:** Gastrointestinal diseases, Medical research

## Abstract

**Background:**

Laparoscopic appendectomy (LA) is a widely used surgical procedure. Patients often suffer from considerable postoperative pain and indigestion, which prolongs their in-hospital stay. Almost 10% of patients develop postoperative complications. The enhanced recovery after surgery (ERAS) program has proven its efficacy in elective surgery and could hypothetically improve LA outcomes. Currently, there is no ERAS program for LA.

**Methods:**

A modified ERAS (mERAS) protocol was studied in a prospective, randomized nonblinded clinical trial. The mERAS group consisted of 50 patients; the control group, of 54 patients. The mERAS protocol included a patient information brochure; minimizing drain use; local anesthesia; low-pressure pneumoperitoneum; early mobilization and oral diet. The primary outcome was postoperative length of stay (pLOS).

**Results:**

Modified protocol reduced median pLOS to 1.25 days vs 2 days in the controls (*p* < 0.0001). Twenty-one (42%) mERAS patients and 4 (7.4%) controls were discharged within 24 h (*p* < 0.001) after surgery; 0 readmissions were reported. Postoperative pain intensity assessed on the visual analogue scale was significantly lower in the mERAS group [mERAS vs control 0 h, 2 h, 6 h, 12 h and 24 h after surgery: 2.33 ± 2.12 vs 4.19 ± 2.08 (*p* < 0.0001), 2.27 ± 1.91 vs 4.02 ± 1.89 (*p* < 0.0001), 2.28 ± 1.98 vs 3.70 ± 1.57 (*p* = 0.0001), 1.98 ± 1.72 vs 3.43 ± 1.54 (*p* < 0.0001) and 1.80 ± 1.74 vs 3.00 ± 1.27 (*p* = 0.032), respectively)]. The severity of shoulder and neck pain was lower but its incidence was similar. Peristalsis recovery was achieved earlier in the study group (median (min–max))—mERAS 7 (2–34) h vs control 11 (3–43) h; p = 0.009) but did not affect the time of the first flatus 23 (2–72) h vs 29 (6–70) h, respectively; *p* = 0.499).

**Conclusions:**

The modified ERAS program for LA has advantages over the traditional approach.

**Registration:**

This trial was registered at ClinicalTrials.gov as NCT03754777 (27/11/2018).

## Introduction

In 2015, over 11.6 million cases of acute appendicitis (AA) were reported worldwide^1^. Unlike open appendectomy, the laparoscopic approach results in less postoperative wound pain, a lower complication rate, and thereby a shorter in-hospital stay^2^. However, considerable postoperative pain and indigestion are often reported after laparoscopic appendectomy^3^, interfering with the quality of rehabilitation and delaying discharge from hospital. Recovery can also be delayed by postoperative complications. Although appendectomy is considered a safe operation, the rate of related complications remains at 10%^4^, with little improvement over the past few years. The postoperative recovery period is still long, averaging 13 days^5^.

ERAS programs have revolutionized perioperative care in elective surgery, and their positive effects are globally acknowledged^6,7^. However, it is uncertain whether ERAS can benefit emergency patients: the literature on this problem is scarce. This is partly due to significant challenges facing the application of enhanced recovery pathways to emergency surgery. For example, a systematic review by Paduraru et al. covered only five eligible studies of ERAS outcomes in emergency surgery. The authors concluded that ERAS programs were safe and feasible for emergency surgery and could reduce the length of in-hospital stay and postoperative complications^8^. Although there have been a few studies showing the benefits of several ERAS components for patients with AA^9–11^, Hamill et al. underlines in his literature review that no enhanced recovery algorithms for AA, as well as evidence-based studies of its effectiveness, have been published so far^3^.

This randomized controlled trial was conducted to evaluate the safety, effectiveness and feasibility of a modified ERAS protocol in patients undergoing LA.

## Materials and methods

### Study design

Our study was a prospective, randomized, nonblinded clinical trial. Patients diagnosed with AA between June 2016 and December 2017 at three medical centers in Moscow, Russia, were consecutively recruited for the study. The patients were provided with comprehensive information about the study, the surgical procedure, postoperative rehabilitation, possible complications, and contact details of the medical and research staff. The simple randomization was made using random numbers generator.

The severity of acute appendicitis was evaluated on the Gomes scale published in 2012^12^. Grades 1 (redness and edema) and 2 (fibrin) were regarded as uncomplicated AA. Grades 3A (segmental necrosis of the appendix with or without perforation), 3B (base necrosis), 4A (abscess), 4B (regional peritonitis), and 5 (diffuse peritonitis) were regarded as complicated AA. The severity of peritonitis was assessed using the Mannheim peritonitis index (MPI).

The following inclusion criteria were applied: American society of anesthesiologists (ASA) classes I and II patients with any grade of AA except 3B (requiring burial of the appendix stump), aged over 18 years.

Exclusion criteria were as follows: refusal to participate in the study or to sign the informed consent form; language barrier; transfer to the intensive care unit (ICU) after surgery; conversion to open procedure; an appendiceal mass found during laparoscopy; pregnancy.

Sample size was calculated after pilot part of study was performed (n = 10), based on difference of length of stay in both groups.

### Randomization

On admission to hospital, the patients were randomly assigned (1:1) to study and control group by a random number generator (Random number generator RNG, Intemodino Group s.r.o). The diagnosis was based on clinical signs, laboratory tests, and ultrasound or computed tomography scans. Treatment allocation was not masked to patients, physicians and researchers.

### Modified ERAS protocol group

As there is no ERAS protocol for AA, we developed a modified ERAS protocol based on the well-known ERAS program for elective (colorectal) surgery. The choice of components was determined by how well they could be adapted to the emergency setting. We hypothesized that patients with AA could benefit not only from the components of ERAS protocol for elective surgery but also from the procedures that had proved to be effective in reducing perioperative stress and improving the quality of rehabilitation. That is why additional intraoperative components were included in our protocol.

#### Preadmission

Not available in the emergency setting.

#### Preoperative care

The patients received a brochure containing a detailed description of their pathology, the surgical procedure, the rehabilitation process, possible complications, etc.

#### Surgery

1. Low-pressure (8–9 mmHg) pneumoperitoneum was established. It is reported that low-pressure pneumoperitoneum is advantageous in reducing the incidence of shoulder and neck pain^13,14^; the ERAS society points to its potential in mitigating the negative impact of CO_2_^15,16^.
2. The appendix mesentery was removed if any signs of its inflammation were detected. Previously, our unpublished pilot study demonstrated that an infected appendix mesentery might be a source of ongoing infection after surgery.
3. Additional local anesthesia. A few studies have demonstrated that intraoperative preemptive intraperitoneal and preperitoneal local anesthesia could relieve postoperative pain and benefit recovery by attenuating stress response^17,18^. Importantly, the last update of the ERAS protocol for elective surgery features regional anesthesia^16^. The mERAS group received 10 ml of 0.25% Ropivacaine solution injected into the space between the internal oblique and transverse abdominal muscles to achieve ilioinguinal and iliohypogastric nerve block; 5 ml of the solution were injected deeper into the preperitoneal space. The subdiaphragmatic space was irrigated and each trocar wound was infiltrated with 5 ml of Ropivacaine at the end of the procedure.
4. Following the concept advocating avoidance of surgical drains learned from ERAS algorithms for elective surgery, a drain was used only in the patients with perforated appendicitis and diffuse peritonitis (Gomes 5)^10,19,20^.

#### Postoperative care

Early mobilization and liquid intake (2 h after surgery) were encouraged; 6 h after surgery the patients were advanced to their regular diet.

### Standard care group

#### Preadmission

Not available in the emergency setting.

#### Preoperative care

The patients were informed about the type of their pathology, the surgical procedure and possible complications. No brochure was given.

#### Surgery

Standard-pressure (12–14 mmHg) pneumoperitoneum was established. Patients with perforated or non-perforated appendicitis complicated by abscess and any type of peritonitis (Gomez ≥ 3A) received a drain. If the appendix mesentery appeared necrotic, it was removed. No intraabdominal anesthesia was performed.

#### Postoperative care

Mobilization was encouraged in 4–6 h; liquids, in 6 h and 12 h, respectively.

### Both groups

#### Preoperative care

Surgery was performed within six hours after admission. Crystalloid isotonic solutions and antibiotic prophylaxis were administered to all patients 30 min before surgery.

#### Surgery

Only experienced surgeons took part in the study. Both groups received balanced general anesthesia; hemodynamics were closely monitored during surgery. LA was performed using monopolar coagulation. The appendix stump was double-ligated with Roeder knots (polyglycolic acid 3/0).

#### Postoperative care

Postoperatively, all patients with complicated appendicitis (Gomez ≥ 3A) were given antibiotics for 3–5 days, depending on their response to therapy. Postoperative pain intensity was measured at rest on the visual analog scale (VAS) immediately after the patients regained responsiveness and then after 2, 6, 12 and 24 h following the surgical intervention. Analgesia (30 mg of Ketorolac) was provided on demand for patients with pain intensity > 5 cm (VAS). Those who developed postoperative nausea and vomiting (PONV) received antiemetics. No intravenous infusions were given postoperatively to any of the patients.

Complications were classified according to the Clavien-Dindo classification^21^. In those patients who had an intra-abdominal drain, the drain was removed 12–24 h after surgery. Intestinal peristalsis was assessed by auscultation every 2 h after surgery.

The patients were discharged home if they had no complications, their VAS score was ≤ 3, they had restored active bowel movement regardless of having flatus or passing stools, and they gave their consent.

A telephone survey was conducted on days 2 and 30 after discharge from hospital. The patients were asked about the presence of pain, the episodes of fever and indigestion, any complications and readmissions.

### Outcomes

The primary outcome of the study was postoperative length of stay (pLOS). The secondary outcomes included the frequency of postoperative complications, the readmission rate, postoperative pain intensity, and the incidence of postoperative shoulder pain.

### Statistics

The obtained data were processed using a commercial Statistica 13.3 software package for Windows (StatSoft Inc., Tulsa, OK). In this article, the results are presented as mean values and standard deviations (SD) for continuous normally distributed variables, as a median (interquartile range (IQR)) for continuous non-normally distributed data, and as counts and percentages for categorical data. Normality of data distribution was tested using the Kolmogorov–Smirnov and Shapiro–Wilk tests. Student’s t test for independent samples was applied to compare continuous variables. Nonparametric quantitative data was assessed using the Mann–Whitney U test. Categorical data and proportions were compared using the Chi-squared test or Fisher's exact test. Differences were considered statistically significant at *p* < 0.05^22,23^. When calculating the required sample size, we drew on the data from our pilot study conducted in January through June 2016, where the mean pLOS of patients with AA was 3.15 ± 2.5 days. The predicted pLOS in the ERAS group was 1.5 days. To bring down the mean pLOS from 3.15 to 1.5 days, with an α error = 0.05 and 1 − β error = 0.90, the t test required a sample size of 50 for each arm.

## Results

**Figure 1:**
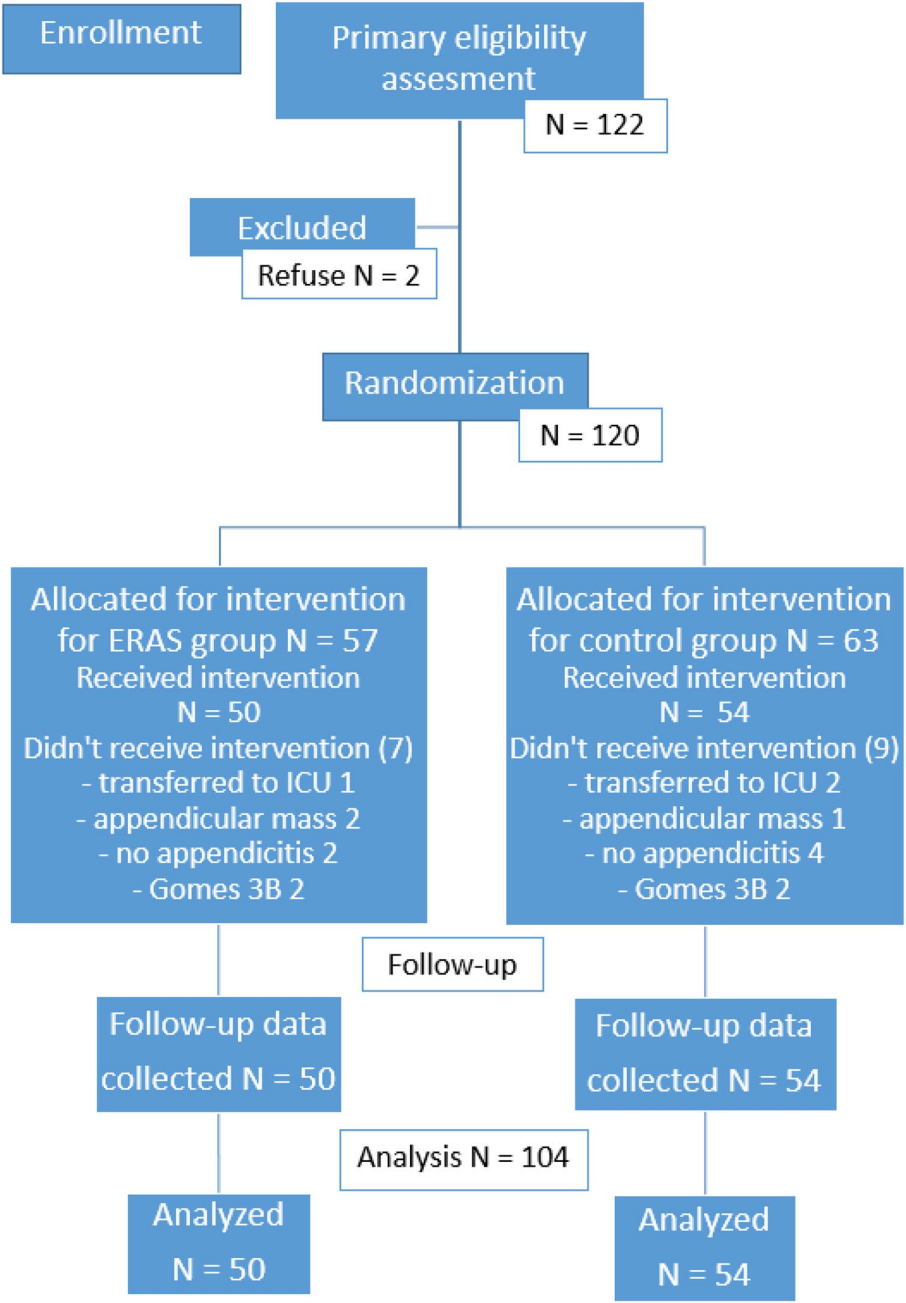
Participant CONSORT 2010 flow diagram.

One hundred twenty-two patients were randomized between June 2016 and December 2017. Eighteen patients were excluded from the study. Details of the enrollment process are shown on the CONSORT 2010 flow diagram (Fig. 1).

Thus, 50 patients in the mERAS group and 54 in the control group were selected for statistical analysis. Baseline characteristics did not differ significantly between the groups (Table 1).

**Table 1:** Patient characteristics.

	mERAS (n = 50)	Control (n = 54)	p
Mean age, years	32.2 ± 9.7	31.4 ± 10.6	0.689
Sex, male	27 (54)	33 (61)	0.590
Comorbidity	11 (22)	4 (7.4)	0.066
Prior abdominal surgery	6 (12)	3 (5.6)	0.413
Uncomplicated appendicitis	39 (78)	41 (76)	0.986
**Complicated appendicitis**			
Gomes 3A	0	4	0.146
Gomes 4A^a^	2	1	0.946
Gomes 4B	2	3	0.930
Gomes 5	7	5	0.653
Complicated appendicitis, total	11 (22)	13 (24)	0.986
MPI^b^	10.0 ± 5.6	8.1 ± 4.7	0.252
Duration of symptoms, h	16 (12–25)	15 (8.5–24)	0.535
Fasting, h	12 (10–15)	12 (10–14)	0.509

Values are presented as mean ± SD, median (IQR) or n (%).

^a^Gomes 3B were excluded from the study.

^b^Mannheim peritonitis index.

The implementation level of each component in mERAS group was as follows: patients informing 100%, local anesthesia 94%, no postoperative antibiotics 92%, no drain 92%, low-pressure peritoneum till the end of the surgery 66%, activation in a range 2 ± 1 h = 56%, liquids in a range 2 ± 1 h 42%, food in a range 6 ± 2 h = 50%. Thus, the lowest implementation level was 42%.

In the mERAS group, the median pLOS was shorter than in the control group (30 h vs. 48 h, *p* < 0.0001). Twenty-one (42%) patients from the mERAS group and 4 (7.4%) patients from the control were discharged within 24 h after appendectomy (OR 9.77, 95% CI 3.06 to 31.2, *p* < 0.0001). The mean duration of appendectomy was similar between the groups: 66.7 ± 23.9 min (mERAS) vs 69.6 ± 25.8 min (control) (*p* = 0.558). The absence of postoperative pain was reported by 13 patients (26%) in the mERAS group and 1 patient (1.8%) in the control group (*p* < 0.0001). In both groups, no patients required opioid analgesics. One patient in the mERAS group and seven patients in the control group developed postoperative complications. All complications were classified as Clavien-Dindo Grade 2. One patient in the mERAS group and one in the control group developed postoperative ileus. Metoclopramide and intravenous infusion of electrolyte solution were started and nasogastric tube was inserted.

Six patients in the control group developed surgical site infections (SSI). Four of them had complicated AA. This complication was accompanied by pain in a rest, an increasing leukocytes level, purulent discharge via drain if it was present and fever (≥ 38 °C) on the postoperative day 1. Palpation in the right ileac region showed tenderness and dense painful mass. All SSI’s were confirmed by ultrasound or abdominal CT. Patients with SSI recovered with antibiotics and NSAIDs.

The results of the study are provided in Table 2.

**Table 2:** Postoperative outcomes.

	mERAS (n = 50)	Control (n = 54)	*P*	Mean difference/odds ratio	95% confidence interval
*Primary outcome*					
**pLOS, days**					
Median (IQR)	1.25 (0.5–2.08)	2 (1.67–4)	< 0.0001		
Mean + SD	1.54 + 1.38	3.25 + 2.76		− 1.71	− 2.58 to − 0.83
*Secondary outcomes*					
VAS^a^ 0 h after surgery^b^, cm	2.33 ± 2.12	4.19 ± 2.08	< 0.0001	− 1.86	− 2.68 to − 1.04
VAS 2 h, cm	2.27 ± 1.91	4.02 ± 1.89	< 0.0001	− 1.75	− 2.49 to − 1.01
VAS 6 h, cm	2.28 ± 1.98	3.70 ± 1.57	0.0001	− 1.42	− 2.12 to − 0.71
VAS 12 h, cm	1.98 ± 1.72	3.43 ± 1.54	< 0.0001	− 1.45	− 2.1 to − 0.81
VAS 24 h, cm	1.80 ± 1.74	3.00 ± 1.27	0.032	− 1.2	− 2.3 to − 0.08
Shoulder pain	18 (36)	29 (53,7)	0.069	0.49	0.22 to 1.07
Shoulder pain, VAS, cm	2.8 ± 0.84	5.0 ± 1.91	0.038	− 2.2	− 4.05 to − 0.35
Complications	1 (2)	7 (13)	0.061	0.14	0.02 to 1.16
Postoperative ileus	1	1	1.00	1.08	0.66 to 17.77
SSI^c^	0	6	0.027		
**Time to mobilization, h**					
Median (IQR)	3 (2–5.5)	6 (4–8)	< 0.0001		
Mean + SD	3.93 + 2.95	7.14 + 4.83		− 3.2	− 4.76 to − 1.65
**Time to the beginning of oral diet, h**					
Median (IQR)	7.5 (4–13)	12 (9–15)	0.001		
Mean + SD	9.03 + 6.11	13.42 + 7.04		− 4.39	− 7.02 to − 1.75
**Peristaltic sound appeared, h**					
Median (IQR)	7 (4.75–11.25)	11 (8–16)	0.009		
Mean + SD	9.78 + 7.84	13.07 + 8.16		− 3.28	− 6.95 to 0.37
**First flatus, h**					
Median (IQR)	23 (10.5–36)	29 (19–40)	0.499		
Mean + SD	26.07 + 16.26	30.33 + 17.09		− 4.26	− 13.9 to 4.67

Values are presented as mean ± SD, median (IQR) or n (%).

^a^Visual analog scale.

^b^Immediately after transfer from operating room.

^c^SSI − organ/space surgical site infection according to CDC classification.

According to the telephone survey, no readmissions, complications or deaths occurred during the 30-day follow-up period in either group.

## Discussion

Numerous studies confirmed the safety of early discharge recommended by ERAS programs for elective surgery and the associated economic benefits^9^.

ERAS protocols were initially developed for colorectal surgery; the studies of their application to this type of surgery still dominate the literature. However, implementation of ERAS principles is reported to improve patient outcomes in other surgical domains as well. Importantly, ERAS algorithms should be regularly updated in response to new data^16^. Our mERAS protocol included components of ERAS guidelines for elective surgery and some methods that could objectively (low-pressure pneumoperitoneum) or presumably (removal of the appendix mesentery) reduce perioperative trauma. In our study, the application of mERAS principles to patients with both uncomplicated and complicated AA undergoing LA resulted in a significant decrease in the median pLOS to 1.25 days and no readmissions in the 30 days following surgery. Numerous studies conducted in the elective surgery setting have proved the safety and feasibility of ERAS programs, as well as their economic benefits^9^. For example, Scott et al. reported that 52.8% of patients were discharged on the day of surgery. In the study by Cross et al., the proportion of such patients was as high as 65%; of them 22.6% were discharged earlier than 7 h after surgery^24,25^. Those studies of uncomplicated appendectomies did not reveal any differences in the readmission and complication rates between patients discharged < 7 and < 24 h after surgery. However, according to Frazee et al., 20% of postoperative complications and 9% of readmissions occurred in patients with perforated appendicitis^11^, in spite of the mean LOS being reduced to 2.67 days. Therefore, although outpatient appendectomy has been practiced for several decades, further research is needed to evaluate its safety in patients with complicated forms of AA^26^.

Our study included both patients with uncomplicated and complicated AA (22% in the mERAS group and 24% in the control group were patients with complicated AA). The implementation of the mERAS program after LA resulted in a decrease in the median LOS to 30 h. Twenty-one (42%) patients in the mERAS group were discharged within 24 h after surgery and 13 (26%) patients, within 12 h; no readmissions or complications were reported.

Pre-emptive local anesthesia was included into ERAS protocols as a stress-mitigating component^16^. In their retrospective study, Foulds et al. demonstrated that additional local anesthesia could shorten LOS in patients with AA^27^. A randomized controlled study by Tranapal et al. revealed a significant reduction in analgesics consumption and postoperative cortisol levels in patients who had received local anesthesia in comparison with the placebo group^18^. The method of local anesthesia proposed in our study benefits patients by reducing postoperative pain and can accelerate recovery in the emergency setting.

Low-pressure pneumoperitoneum is regarded by the ERAS Society as a promising approach that could enhance patient rehabilitation^15^. According to the systematic review of the Cochrane database, the use of low intraabdominal pressures during laparoscopic cholecystectomy results in reduced intensity and incidence of neck and shoulder pain in comparison with standard CO_2_ pressure^13^. Therefore, the tendency to lower incidence of neck and shoulder pain and the significant difference in its intensity observed in our study may be associated with the effect of low-pressure pneumoperitoneum. Moreover Matsuzaki et al. shows that low-pressure laparoscopy may decrease local inflammation, resulting in better clinical outcomes. Both with warmed, humidified gas it significantly lowered expression of inflammation-related genes in peritoneal tissues compared to the standard conditions. Besides, authors showed the low-pressure laparoscopy and humidified gas independently decreased the likelihood of a high VAS pain score (> 30) after surgery^28^.

Early oral diet and early mobilization are well-known components of ERAS pathways for elective surgery; they improve gastrointestinal motility, prevent postoperative ileus and anastomosis dehiscence^7,15,16^. However, the adoption of these key components by emergency surgery and the assessment of their effectiveness are facing difficulties. In our study, time to mobilization and oral food intake was longer than expected in both groups because some patients were admitted to hospital at nighttime. So for example, in mERAS group the average percentage of implementation for early activation was 56%, while for patients operated in the daytime it was 63%. Gastrointestinal function recovery appeared to take less time in the mERAS group, but the time of the first flatus was similar between the groups. Perhaps, AA and LA themselves are not accompanied by significant inhibition of bowel motility.

The ERAS Society guidelines for elective surgery recommend minimizing abdominal drain use. The World Society of Emergency Surgery (WSES) guidelines do not contain strict recommendations on drainage practices in complicated AA due to the lack of robust evidence in the literature, but suggest that drains should be used with caution^19^. In our study, preemptive drainage did not prevent postoperative organ/space SSI in patients from the control group.

In our study, the appendix mesentery was removed in patients from the mERAS group. This could positively contribute to the reduction of SSI. However, the influence of this element on recovery and complications is only a hypothesis, and it requires further investigation in experimental and clinical studies to identify its potential benefits and harms.

We evaluated the mERAS program as a single algorithm, which could be a limitation of our study. It is difficult to assess the impact of each individual component on postoperative recovery. The range of components borrowed from the ERAS programs for elective surgery varies between the studies of their application to emergency surgery due to significant challenges facing their adaptation to the emergency setting. It was reported that compliance with the protocol was 57% for emergency surgery compared with 77% for elective surgery^29^. In our study, we were able to implement 42% (fluid intake within 2 h) to 92% (avoidance of drain use) of all ERAS recommendations (the mean value was 72%). So far, it remains unclear which components of the program make the greatest contribution to the recovery process in urgent care. Further research is needed to clarify what affects patient outcomes the most: the benefit of individual components of the program or the application of the program in its entirety^8^.

Another limitation of our study was the primary focus on the components directly related to surgery and early postoperative period. The emergency setting makes preoperative care impossible and long-lasting postoperative rehabilitation unnecessary in the majority of LA cases^24, 25^. The exclusion of patients transferred to the ICU may have contributed to the favorable outcome of the study. The presence of additional factors affecting the quality of rehabilitation, including the use of catheters, nasogastric tubes, the need for prolonged intravenous infusion and parenteral feeding, and small number of patients in ICU, did not allow us to analyze this subgroup.

## Conclusions

The modified enhanced recovery program for emergency LA results in early patient discharge, less postoperative pain and benefits patients without compromising other outcomes.

### Ethical statement

All procedures performed in studies involving human participants were in accordance with the ethical standards, all experimental protocols were approved by the Pirogov Medical University local ethics committee (N165-22-05) and comparable ethical standards and registered at ClinicalTrials.gov as NCT03754777 (27/11/2018). All patients gave informed consent to participate in the study.
