# Author Correction: Evaluation of enhanced recovery after surgery program components implemented in laparoscopic appendectomy: prospective randomized clinical study

**DOI:** 10.1038/s41598-021-97902-3

**Published:** 2021-09-14

**Authors:** Taras Nechay, Alexander Sazhin, Svetlana Titkova, Alexander Tyagunov, Mikhail Anurov, Kirill Melnikov-Makarchuk, Anton Tyagunov

**Affiliations:** Pirogov Russian National Research Medical University, Moscow, Russia

Correction to: *Scientific Reports* 10.1038/s41598-020-67591-5, published online 01 July 2020

In the original version of this Article Taras Nechay, Alexander Sazhin and Alexander Tyagunov were incorrectly affiliated with ‘Pirogov Russian National Research Medical University, State Clinical Hospital #1, Moscow, Russia’. Kirill Melnikov-Makarchuk and Anton Tyagunov were incorrectly affiliated with ‘Pirogov Russian National Research Medical University, State Clinical Hospital Named After V.M. Buyanov, Moscow, Russia.’

The correct affiliation for all authors is listed below.

Pirogov Russian National Research Medical University, Moscow, Russia.

The original Article has been corrected.

